# Research progress on nanotoxicity and detoxification of cobalt in metal-based implants

**DOI:** 10.1080/07853890.2025.2532120

**Published:** 2025-07-11

**Authors:** Wentao Fan, Mingxin Guo

**Affiliations:** The Affiliated Yixing Hospital of Jiangsu University, Yixing, China

**Keywords:** Metal-based implants, particles, cobalt nanoparticles, joint arthroplasty, toxicity, detoxification, ferroptosis

## Abstract

**Background:**

Cobalt nanoparticles (CoNPs), a primary wear debris from joint prostheses, exhibit significant cytotoxicity. This review aims to synthesize current understanding of CoNPs’ toxicity mechanisms, their local and systemic pathological effects, the emerging role in ferroptosis, potential detoxification strategies, and outline critical future research directions.

**Discussion:**

CoNPs are internalized by cells, primarily *via* phagocytosis, and undergo lysosomal degradation. This process triggers substantial reactive oxygen species (ROS) generation, a key mechanism driving cytotoxicity and cell death pathways. Locally, at the implant site, CoNPs contribute to adverse outcomes including aseptic inflammation, osteolysis, and inflammatory pseudotumor formation. Furthermore, systemically disseminated CoNPs pose risks to distant organs, with potential impairments reported in liver, kidney, cardiac, thyroid, and neurological functions. Emerging evidence specifically implicates CoNPs in inducing ferroptosis, an iron-dependent form of regulated cell death. Current research explores various strategies for mitigating CoNPs toxicity.

**Conclusions:**

CoNPs released from joint prostheses present substantial local and systemic health hazards. Their toxicity is primarily mediated through lysosomal degradation, ROS overproduction, and the induction of cell death, including ferroptosis. While mechanisms are increasingly understood, further research is crucial to fully elucidate the long-term systemic consequences and to develop effective clinical detoxification and preventative strategies for patients with cobalt-containing implants.

## 1. Introduction

First-generation metal-on-metal total hip arthroplasty (MOM-THA) devices emerged in the 1950s [1]. Within a decade, however, MOM-THA was largely superseded by metal-on-polyethylene total hip arthroplasty (MOP-THA), which demonstrated lower friction coefficients and superior wear resistance [2]. Subsequent developments introduced low-wear ceramic prostheses. Nevertheless, cobalt-alloy implants retained significant clinical relevance due to technical limitations and fracture risks associated with early ceramic components. Post-implantation, all artificial joint components experience wear through mechanical stress, micromotion, and fatigue corrosion, releasing particulate debris of varying diameters and metal ions. These wear particles trigger adverse local tissue reactions (ALTR) and may induce systemic multiorgan toxicity, including hypertrophic cardiomyopathy, polycythemia, thyroid dysfunction, hearing loss, and visual impairment [3].

In alloy prostheses, cobalt (Co) serves as the primary component, making cobalt nanoparticles (CoNPs) the most prevalent and toxic wear debris from these implants [4]. Clinically, CoNPs contribute significantly to premature joint failure [5]. Metallic wear debris occurs across all bearing surfaces—including metal-on-metal (MOM), ceramic-on-ceramic, and metal-on-polyethylene (MOP) interfaces—with even ceramic hip replacements associated with fatal cobalt toxicity in reported cases [6]. Rising clinical reports of adverse reactions to metallic debris have heightened clinician vigilance. Notably, cobalt toxicity manifestations extend beyond established clinical symptoms, as many arthroplasty patients report unexplained systemic complaints potentially linked to cobalt exposure. In 2010, Tower comprehensively documented systemic complications following MOM arthroplasty, establishing the diagnostic concept of arthroprosthetic cobaltism [7]. This syndrome has gained substantial recognition, with subsequent studies confirming its occurrence not only in MOM bearings but also with other interface types [8]. Crucially, wear or corrosion-derived particles from prosthetic components induce both local adverse biological reactions and systemic multiorgan toxicity, thereby increasing revision surgery risk [9].

Ferroptosis has recently emerged as a distinct form of regulated cell death, mechanistically divergent from autophagy and apoptosis. This process is primarily defined by iron-dependent hydroxyl radical generation through Fenton reactions, resulting in reactive oxygen species (ROS) accumulation, depletion of reduced glutathione (GSH), and profound suppression of glutathione peroxidase 4 (GPx4) activity—collectively culminating in cell death [10]. Significantly, studies reveal that CoNPs internalized *via* endocytosis likewise trigger excessive ROS through Fenton chemistry while simultaneously depleting GSH and inhibiting GPx4 activity, driving both cytotoxicity and genotoxicity [11]. Given this mechanistic overlap with ferroptosis hallmarks, investigating CoNPs toxicity through the ferroptosis paradigm offers a promising research avenue for identifying novel therapeutic targets.

This review examines metallic implant-derived wear debris *in vivo*, focusing particularly on CoNPs as the most biologically aggressive particulate species. We analyze CoNPs generation mechanisms, recent advances in understanding their local/systemic toxicity, and provide a critical synthesis of detoxification challenges and future directions.

## 2. Mechanism of toxicity generation of CoNPs

Total joint arthroplasty—a globally established intervention for end-stage joint pathologies including osteoarthritis, avascular necrosis, and select femoral neck fractures—effectively alleviates pain, restores function, and improves quality of life [12]. However, long term wear and corrosion at prosthesis interfaces persistently generate metallic particles of varied diameters and morphologies. Among these, nanoscale cobalt (Co) and chromium (Cr) nanoparticles predominate and have garnered significant research attention [13]. Their high surface-area-to-mass ratio confers enhanced biological reactivity, increased toxicity, and efficient systemic dissemination. This section examines CoNPs toxicity through four interconnected mechanisms, as summarized in Table 1.

**Table 1.** Mechanism of toxicity generation of CoNPs.

Type	Description
Mechanism of wear debris generation	Prosthesis reduction status directly modulates MOM wear: well-reduced implants lower wear rates, while poor reduction increases debris generation. Debris size/roughness affects inflammation, and gender may influence metal ion kinetics.
Inflammation and injury mechanism	CoNPs from MOM prostheses trigger macrophage infiltration/apoptosis, releasing metal ions and elevating ROS. This cascade induces muscle atrophy and cell death *via* HIF-1α activation and autophagy dysregulation, driving periprosthetic tissue damage. Concurrently, T/B cell infiltration exacerbates inflammation, promoting osteolysis and apoptosis.
Local and systemic adverse reactions	CoNPs can trigger local inflammation and complications, such as ALVAL, and may even induce systemic reactions *via* hematogenous dissemination.
Genetic toxicity	CoNPs induce osteolysis and tissue destruction, with potential genotoxicity/DNA damage. Elevated Co/Cr-mediated ROS provoke DNA damage and multi-organ disorders, contraindicating MOM arthroplasty in renal failure patients.

### 2.1. Mechanisms of wear debris generation

Similar to other joint prostheses, MOM bearings inevitably generate wear debris during articulation. Studies indicate that well-reduced anatomic positioning correlates with lower wear rates, primarily yielding Cr_2_O_3_ particles (<100 nm) and reduced *in vivo* Co^2+^ concentrations. Conversely, malreduced prostheses demonstrate wear rates up to 100 mm³/year, producing larger metallic particles and significantly elevated Co^2+^ levels [14]. *In vitro* simulations confirm that edge-loading articulation dramatically increases particle generation during translation/rotation [15]. This promotes localized accumulation of metallic debris and exacerbates inflammatory tissue responses, ultimately triggering complications including aseptic loosening, pseudotumors, and aseptic lymphocyte-dominated vasculitis-associated lesions (ALVAL) [16]. Consequently, implant positioning—particularly edge loading—critically regulates metallic debris generation.

The physical characteristics of wear debris significantly influence the biological response. Studies on cemented/cementless prostheses indicate that particles of different compositions elicit similar inflammatory reactions, suggesting that size and load may be more critical determinants of periprosthetic osteolysis than chemical composition [17]. Larger, irregular particles substantially elevate interleukin-1β levels through lysosomal damage and inflammasome activation, whereas smooth spherical particles lack this effect but exacerbate lysosomal instability, leading to cellular injury [18]. However, controversy persists regarding the inflammatory impact of particle physical properties. This is partly because *in vitro* Co and Cr inadequately replicate the complex *in vivo* wear environment [19], and the ‘biological persistence’ of particles can not be reproduced by simulators *in vivo* [20]. Sex also influences metal ion dynamics. Female patients with MOM-HRA exhibit a higher risk of elevated blood metal ion levels, whereas male patients show higher periprosthetic particle loads 9 months postoperatively [21,22]. This suggests sex may modulate the generation and persistence of metal debris, though the precise mechanisms require further investigation. Smaller female acetabular components increase edge-loading risk, accelerating Co release, and faster Co clearance in females *vs*. prolonged Cr retention. Current evidence remains inconclusive due to heterogeneous sampling protocols across studies.

### 2.2. Inflammation and injury mechanism

Crucially, the long term accumulation of metal nanoparticles triggers multilayered pathological cascades. Ricciardi et al. found that MOM-RHA and MOM-THA showed more macrophage infiltration and death than non-MOM-THA, indicating that metal ion release may be caused by the widespread phagocytosis/endocytosis of metal nanoparticles [23]. Additionally, this procedure raises ROS significantly, which encourages macrophage death. Because it involves the buildup of CoNPs and the ongoing reactive response of macrophages, this histological pattern takes a long time to become apparent. According to published research, CoNPs can mimic hypoxia by stabilizing transcription and activating hypoxia-inducible factor (HIF), which may be a major contributor to the possible harm to peri-prosthetic tissues [15]. CoNPs can cause muscle atrophy and harm to the viability of C2C12 myotube cells through Co generated by macrophages. This can result in cell death, which is regarded as a particular type of programmed necrosis linked to apoptosis. This is brought on by activating death receptors like TNF-related apoptosis-inducing ligand (TRAIL), Fas ligand, and tumor necrosis factor *ɑ* (TNF-*ɑ*). The autophagic degradation system is the main cause of morphological atrophy. Although it helps to remove and remodel damaged materials [24], it also causes early activation of the NF-*қ*B pathway, increased expression of TNF-*ɑ* and other pro-inflammatory chemokines, and sustained autophagy through delayed changes in phosphatidylinositol 3-kinase/protein kinase B levels [25]. Furthermore, certain proteasomes linked to E3 ubiquitin ligase also contribute to the autophagic system’s activation, which eventually results in chronic skeletal muscle atrophy.

Recent research has shown that titanium oxide nanoparticles cause cells to undergo a dose-dependent autophagic response. In the meanwhile, methionine can be oxidized by nickel nanoparticles, which results in the buildup of altered proteins and inhibits autophagy [26]. This offers a foundation and guidance for comprehending how titanium and nickel metals work to prevent autophagy in upcoming metal-on-metal joint replacements. But it’s important to consider the possible drawbacks of CoNPs-induced autophagy, such as causing osteoblasts to undergo apoptosis, which might possibly be connected to wear-induced osteolysis [27]. Furthermore, in histological investigations, Liu et al. noted cell-mediated immune reactions around the prosthesis. Diffuse infiltration of T, B, and plasma cells; perivascular infiltration; the development of high endothelial venules; extensive fibrin exudation; the accumulation of macrophages with droplet-like inclusions; and infiltration by necrotic cells and eosinophils were among the histological features. According to the immunological profile of the infiltrating cells, CD8^+^ T cell predominate and are found in the peri-prosthetic tissues. This suggests that cell-mediated immune responses have a role in the processes of inflammation and damage [28].

### 2.3. Local and systemic adverse reactions

Critically, wear-derived CoNPs induce not only local inflammatory complications predictive of implant failure, but also systemic dissemination with potentially subclinical toxicity. These particles, released from metal-based implants, trigger inflammation in surrounding tissues, resulting in local complications, such as aseptic loosening, pseudotumors, and ALVAL. Moreover, they can enter the systemic circulation, causing systemic reactions that include neurological and organ pathologies. Notably, ALVAL serves as an early indicator of lymphocytic neogenesis and is a key predictor of subsequent tissue necrosis and implant failure following joint replacement [16]. Analysis of biopsy tissues from 123 failed joint replacements by the Natu team revealed distinct histological changes, including varying degrees of superficial necrosis, aggregates of metal-debris-laden macrophages, and progressive ALVAL featuring germinal centers. The study further demonstrated that MOM-associated ALVAL is distinguished from other arthropathies by key features, macrophage-mediated extensive necrosis, architectural destruction, and prominent metal debris deposition [29]. Perivascular infiltration of diverse T-lymphocyte subsets was observed in the tissues; the pro-inflammatory cytokines released by these cells can activate osteoclasts, accelerating osteolysis and ultimately leading to ALTR. Additionally, CoNPs can disseminate to systemic organs, such as bone marrow, blood, and liver [30]. It is crucial to emphasize that although some patients with elevated blood cobalt levels remain clinically asymptomatic with no measurable adverse effects, the potential risk of systemic cobalt toxicity warrants vigilant monitoring. Specialized studies have been conducted on metal particle levels with the aim of establishing predictive thresholds for implant failure, which is crucial for the clinical management of MOM patients. While epidemiological studies have not found definitive toxic effects of MOM on the kidneys, heart, or nervous system, some individual cases of implant failure show that metals may induce cardiomyopathy or thyroid/nerve damage, and this damage may persist even after the prosthesis is removed [31].

### 2.4. Genetic toxicity

Particularly relevant for younger patients, emerging evidence reveals systemic cobalt toxicity extends beyond local damage to include genotoxic risks and placental transfer, necessitating heightened clinical vigilance. CoNPs are well known for their genotoxic effects. The primary mechanisms of cobalt’s genotoxicity include single-strand breaks and crosslinking [32]. Existing evidence suggests no definitive association between Co and Cr levels and chromosomal abnormalities, nor have such abnormalities demonstrated carcinogenicity. Although metal ions theoretically pose teratogenic risks, this remains unconfirmed due to insufficient clinical data. Co and Cr can penetrate biological barriers to mediate signal transduction and induce irreversible DNA alterations. In a comparative study of mouse fibroblast responses to cobalt, CoCl_2_ primarily caused DNA damage, while CoNPs induced cellular transformation and genotoxicity. Given the prevalent use of MOM implants in younger patients, placental transfer risks during pregnancy require consideration. Research confirms significantly elevated Co and Cr levels in umbilical cord blood of pregnant women with MOM implants relative to controls [33], confirming transplacental passage of circulating metal ions and resulting fetal exposure to potential teratogens. Consequently, women of reproductive age should be counseled regarding these risks before MOM implantation.

Co and Cr generate ROS, increasing free radical concentrations while depleting antioxidants. When ROS formation exceeds the clearance capacity of antioxidant systems, it induces DNA damage. This leads to impaired DNA repair mechanisms, sister chromatid exchange, and aneuploidy [24]. Elevated ROS also promotes lipid peroxidation and contributes to diverse pathological effects, including chronic inflammation, cardiovascular diseases, and cancer.

Although cobalt forms stable oxides less readily and undergoes more efficient metabolic clearance than chromium, the long-term wear of MOM implants results in continuous cobalt release. Consequently, MOM joint replacements are generally contraindicated in patients with chronic renal failure. Furthermore, renal impairment elevates systemic Co and Cr levels—a significant clinical concern. These patients require thorough evaluation before considering metal-based implants [34].

## 3. Research on detoxification of CoNPs

The cytotoxicity and genotoxicity of CoNPs have been widely recognized, but the specific molecular mechanisms remain unclear. The research results are summarized in Table 2.

**Table 2.** Toxicity mechanism of CoNPs.

Type	Mechanisms
Oxidative stress	Oxidative stress is currently recognized as the primary toxicity mechanism of CoNPs. CoNPs generate excessive ROS through catalytic Fenton-like reactions. This ROS surge depletes GSH, triggering lipid peroxidation, protein oxidation, and mitochondrial impairment. Subsequent cytoskeletal disruption leads to chromosomal condensation and aberrations, ultimately causing organelle damage, cellular apoptosis, and cell death. This cascade is closely linked to dysregulation of the Keap1-Nrf2-ARE signaling pathway.
Hypersensitivity	Type IV hypersensitivity may serve as the pathogenic contributor to both local and systemic adverse reactions elicited by MOM prostheses *in vivo*
Competition and replacement	Co^2+^ disrupt metalloenzyme function and calcium homeostasis through irreversible binding and competitive inhibition, ultimately inducing dysregulation of cellular signaling pathways.
Endoplasmic reticulum stress and autophagy	Wear particles from metallic implants promote inflammatory factor expression by inducing endoplasmic reticulum (ER) stress, while simultaneously activating autophagy-mediated osteoblast apoptosis. Conversely, inhibition of both autophagy and ER stress reduces inflammatory secretion and suppresses osteoclastogenesis, thereby alleviating osteoblast damage

### 3.1. Oxidative stress

Oxidative stress (OS) is widely recognized as the primary toxic mechanism of CoNPs. OS arises from an imbalance between oxidation and antioxidation within the organism. This imbalance alters protease activity, generates abundant ROS as oxidative intermediates, and causes consequent cellular damage [35]. Furthermore, OS is implicated in the pathogenesis of numerous diseases. Substantial research confirms that CoNPs induce elevated cellular OS, leading to excessive ROS production. This, in turn, triggers toxic damage to various cell types and their DNA [36].

Studies demonstrate that CoNPs can enter cells through endocytosis or pinocytosis. Their toxic effects depend on intracellular degradation and the microenvironment within cells, particularly pH. After cellular uptake, CoNPs generate excessive ROS *via* a Fenton-like reaction. This ROS depletes GSH, induces lipid peroxidation and protein oxidation, causes mitochondrial damage, disrupts the cytoskeleton, and leads to chromosomal condensation and aberrations. Ultimately, this cascade results in organelle damage, apoptosis, and cell death [37].

### 3.2. Hypersensitivity

Co, Cr, and Ni are among the metals known to cause immunological sensitization. Metal ion hypersensitivity may be linked to both local and systemic adverse responses caused by joint prostheses *in vivo*. Studies indicate that individuals undergoing total hip replacements exhibit a higher incidence of metal hypersensitivity and postoperative lymphopenia compared to the general population. In ELISpot studies and lymphocyte transformation tests, long-term exposure to micrometer-sized Co-Cr particles has been shown to induce immunological sensitization in mice. Additionally, the Th1-driven immune response to these particles suggests that type IV hypersensitivity contributes to the disease pathogenesis [15,38].

### 3.3. Competition and replacement

Various metal-containing proteins can interact with Co^2+^, which may displace their intrinsic metal ions, impairing or abolishing the protein’s original function. Several studies indicate that Co^2+^ primarily disrupts steroidogenesis and neuromuscular transmission by inhibiting Ca^2+^ channels, thereby disturbing Ca^2+^ homeostasis. Simonsen et al. demonstrated that Co^2+^ and Ca^2+^ compete for Ca^2+^-binding sites on the carrier protein responsible for maintaining Ca^2+^ balance in erythrocytes. This competition reduces intracellular Ca^2+^ levels and may compromise Ca^2+^ signaling. Their experiments further revealed that Co^2+^ binding to cytosolic proteins is essentially irreversible. According to *in vitro* studies, Co^2+^ binds to Ca^2+^-ATPase in the sarcoplasmic reticulum and inhibits Ca^2+^ inflow by blocking Ca^2+^ binding to voltage-dependent Ca^2+^ channels [39–41].

### 3.4. Endoplasmic reticulum stress and autophagy

*In vivo*, wear particles generated by metal-based implants can induce endoplasmic reticulum stress, which subsequently stimulates the production of multiple inflammatory cytokines. Research further demonstrates that autophagy mediates osteoblast apoptosis triggered by these wear particles. Critically, inhibiting both autophagy and endoplasmic reticulum stress concurrently suppresses osteoclastogenesis and reduces inflammatory cytokine release, thereby mitigating the detrimental effects of wear particles on osteoblasts [42,43].

## 4. Etoxification strategies for CoNPs

Reported serum Co^2+^ levels following MOM arthroplasty vary substantially across studies, ranging from 0.9 to 6521 μg/L. Currently, no definitive evidence establishes a clear correlation between blood Co^2+^ levels and clinical toxicological symptoms [30]. The UK Medicines and Healthcare products Regulatory Agency (MHRA) recommends adopting 7 μg/L as a reference threshold for clinical decision-making related to cobalt. However, this threshold is based on reference ranges in the general population rather than comparative data from symptomatic and asymptomatic patients. The US Food and Drug Administration (FDA) has not defined a specific threshold but recommends serial monitoring of metal ion levels in symptomatic patients. The FDA emphasizes that blood Co^2+^ concentrations must be interpreted in the context of the patient’s symptoms, baseline renal function, and potential alternative sources of exposure [44,45].

Currently, there is no widely accepted specific therapy for treating cobalt toxicity. In patients with persistently elevated blood Co^2+^ levels and associated symptoms following arthroplasty, levels typically decline after prosthesis removal; however, symptoms often do not fully resolve [46]. *In vivo* studies exploring chelation therapy with agents like ethylenediaminetetraacetic acid (EDTA) and 2,3-dimercaptopropane-1-sulfonate (DMPS) demonstrated reductions in Co^2+^ levels, yet symptomatic improvement was not significant. Consequently, prosthesis removal remains necessary [47,48]. For Co toxicity-related complications, such as hypothyroidism, cardiomyopathy, and pulmonary disease, management primarily involves symptomatic treatment, including thyroxine replacement therapy, corticosteroids, and angiotensin-converting enzyme inhibitors [49]. Therefore, for cobalt toxicity originating from prosthetic joint replacement, the cornerstone of treatment remains removal of the prosthesis, supplemented by symptomatic management. Although isolated case reports describe the use of hemodialysis in single sessions, its therapeutic efficacy remains unclear.

As shown in Table 3, several substances have been found to be effective in reducing the cytotoxicity and genotoxicity caused by CoNPs in *in vitro* studies. These include antioxidants (N-acetylcysteine (NAC), melatonin, ascorbic acid, astaxanthin, and α-tocopherol), antagonists (zinc ions), chelating agents (EDTA), DMPS, and compounds used in traditional Chinese medicine (salidroside) [50–53]. Although studies looking into detoxification strategies specifically for CoNPs toxicity have not yet been published, studies using a variety of cell types have consistently shown that these agents exhibit varying degrees of protective effects against CoNPs-induced cytotoxicity *in vivo*. Antioxidants are the most widely accepted class of detoxifying agents.

**Table 3.** Detoxification mechanisms.

Agent	Mechanism
N-acetylcysteine	↑GSH synthesis; Direct ROS scavenging
Melatonin	↑Endogenous antioxidants; Mitochondrial protection
Ascorbic acid	Electron donation; Regenerate oxidized GSH
Astaxanthin	Quench singlet oxygen; ↓Lipid peroxidation
α-Tocopherol	Lipid-soluble antioxidant; ↓Membrane damage
DMPS	Co²⁺/Cr³⁺ chelation → renal excretion
Salidroside	↓ROS-induced apoptosis; ↑HO-1 expression

## 5. The relationship between CoNPs and ferroptosis

The toxicity of CoNPs stems primarily from their rapid dissolution into Co^2+^ ions following cellular uptake. This is supported by experimental evidence confirming the significant cytotoxicity of CoCl_2_ and observations demonstrating rapid dissolution of CoNPs in cell culture [54]. Significantly, Gupta et al. discovered that CoNPs—unlike nanoparticles derived from other cemented carbides—induce ferroptosis-like cell death in models of neurodegenerative disease. This finding provides a novel perspective on the mechanisms underlying Co^2+^-mediated cytotoxicity [55].

Research has revealed distinct characteristics in CoNPs-induced cell death. Following cellular uptake of CoNPs, cell display Ca^2+^ influx, mitochondrial swelling, and substantial formation of hydroxyl radicals, ultimately leading to cell death [56]. Notably, this process is not inhibited by apoptosis or necroptosis inhibitors but is mitigated by the ferroptosis inhibitor Liproxstatin-1 or the iron chelator deferoxamine, suggesting CoNPs trigger a ferroptosis-like response. Cell death caused by cobalt toxicity shares key features with ferroptosis, as both involve elevated intracellular ROS levels, increased lipid peroxidation, GSH depletion, markedly reduced GPx4 activity, and activation of the Nrf2 signaling pathway. Furthermore, antioxidants, such as NAC and α-tocopherol mitigate the effects of both conditions [57,58]. As summarized in Table 4, CoNPs exposure induces an ROS surge and GSH depletion, though quantitative thresholds need further validation. These findings not only confirm that CoNPs toxicity arises from the activation of oxidative stress pathways but also offer novel insights for investigating the link between CoNPs and ferroptosis, meriting deeper investigation.

**Table 4.** Proposed biomarker responses to CoNPs exposure (limited direct data).

Biomarker	Acute exposure trend	Chronic exposure trend
ROS	Robust increase	Sustained elevation
GSH	Depletion observed	Progressive decline
GPx4	Context-dependent	Proposed inhibition

## 6. Discussion

The proposal of MoM-THA has benefited thousands of patients, and their pain and quality of life have been improved. However, the wear and tear problems caused by it can’t be ignored. Given the projected increase in THA procedures over the coming decades and the continued widespread clinical use of cobalt-based alloys, these issues are likely to become increasingly prevalent in clinical practice. The majority of contemporary THA implants are constructed from Ti-alloys at present, which are generally believed to generate fewer ALTR compared to CoCr-alloys [59]. The precise molecular mechanisms underlying CoNPs-induced cytotoxicity, genotoxicity, and inflammatory cytokine release remain incompletely defined, coupled with a scarcity of definitive detoxification strategies and effective antidotes; current clinical management remains limited to prosthesis removal and symptomatic treatment. Presently, potential adverse outcomes include localized tissue damage, systemic complications arising from metal particle exposure, and revision surgery rates exceeding initial projections. Consequently, developing targeted solutions to mitigate CoNPs toxicity represents a major research priority.

Regarding elevated metal particle levels and aseptic loosening induced by wear, revision surgery currently constitutes the primary intervention. Considering treatment costs, utilizing ceramic-on-metal bearing surfaces may offer improvements. Furthermore, with an increasing number of younger patients undergoing THA, particular attention must be paid to the potential impact of elevated metal ion levels in young women, especially during pregnancy, on fetal development, as studies indicate women exhibit a greater propensity for elevated metal ion concentrations. Research on CoNPs toxicity remains predominantly *in vitro*. However, numerous factors significantly influence the wear rate and physicochemical properties of debris generated by simulators, including cycle count, lubricant composition, implant diameter, and surface topography. More extensive *in vivo* investigations are warranted. Additionally, the synovial fluid within joints, containing proteins and salts, substantially alters the chemical surface of implants, thereby profoundly affecting the physicochemical characteristics of wear debris. Future toxicological studies must incorporate these factors to better understand particle generation, size distribution, and chemical alterations in both malpositioned and well-functioning implants, ultimately elucidating their potential contribution to inducing local and systemic adverse effects.

Critical insights from murine models illuminate CoNPs toxicity pathways, with demonstrating that *in vivo* CoNPs exposure triggers progressive osteolysis and soft-tissue necrosis mirroring human ALVAL histopathology. These findings reveal time-dependent CD3+ T cell perivascular aggregation and Th1-dominant cytokine dysregulation, alongside a dose-response relationship wherein 500 ppm CoNPs induced triple the osteoclast activation *versus* controls. Mechanistically, murine TNF-*α*/NF-*κ*B overactivation provides biological validation for CoNPs-driven implant failures in humans, directly supporting clinical strategies for high-risk patient monitoring based on blood metal thresholds. Furthermore, this model establishes a platform for preclinical testing of chelation or antioxidant therapies. Murine cobalt clearance rates exceed human metabolic capacity by 8-fold, suggesting chronic toxicity thresholds may differ across species. Future studies should therefore prioritize longitudinal exposure designs and genetically diverse models to bridge these interspecies gaps while reinforcing clinical relevance.

Antioxidants and metal ion antagonists remain the primary focus in detoxification research targeting CoNPs toxicity. However, current evidence is limited to preclinical *in vivo* studies, and widely accepted detoxifying agents or therapeutic approaches are lacking. Research into novel cell death mechanisms, such as ferroptosis, offers promising new directions for advancing our understanding of cobalt toxicity and developing targeted detoxification strategies. Ultimately, two core strategies exist for reducing metallic implant toxicity in patients: developing novel biocompatible implant materials or discovering effective detoxifying agents and treatment protocols.

## 7. Conclusion

CoNPs mediate periprosthetic tissue damage through macrophage apoptosis, HIF-1*α* activation, and autophagic dysregulation, with systemic dissemination posing significant teratogenic risks. The field now faces three critical unmet needs: establishing standardized Co-Cr toxicity thresholds, developing predictive *in vivo* models reconciling species-specific metabolic variations, and clinically validating targeted antioxidant therapies. Addressing these gaps will enable safer implant strategies and evidence-based patient management.

## Data Availability

Data sharing is not applicable to this article as no data were created or analyzed in this study.
